# Are deep models in radiomics performing better than generic models? A systematic review

**DOI:** 10.1186/s41747-023-00325-0

**Published:** 2023-03-15

**Authors:** Aydin Demircioğlu

**Affiliations:** Institute of Diagnostic and Interventional Radiology and Neuroradiology, University Hospital Essen, Hufelandstraße 55, 45147 Essen, Germany

**Keywords:** Artificial intelligence, Deep learning, Machine learning, Radiology, Radiomics

## Abstract

**Background:**

Application of radiomics proceeds by extracting and analysing imaging features based on generic morphological, textural, and statistical features defined by formulas. Recently, deep learning methods were applied. It is unclear whether deep models (DMs) can outperform generic models (GMs).

**Methods:**

We identified publications on PubMed and Embase to determine differences between DMs and GMs in terms of receiver operating area under the curve (AUC).

**Results:**

Of 1,229 records (between 2017 and 2021), 69 studies were included, 61 (88%) on tumours, 68 (99%) retrospective, and 39 (56%) single centre; 30 (43%) used an internal validation cohort; and 18 (26%) applied cross-validation. Studies with independent internal cohort had a median training sample of 196 (range 41–1,455); those with cross-validation had only 133 (43–1,426). Median size of validation cohorts was 73 (18–535) for internal and 94 (18–388) for external. Considering the internal validation, in 74% (49/66), the DMs performed better than the GMs, vice versa in 20% (13/66); no difference in 6% (4/66); and median difference in *AUC* 0.045. On the external validation, DMs were better in 65% (13/20), GMs in 20% (4/20) cases; no difference in 3 (15%); and median difference in *AUC* 0.025. On internal validation, fused models outperformed GMs and DMs in 72% (20/28), while they were worse in 14% (4/28) and equal in 14% (4/28); median gain in AUC was + 0.02. On external validation, fused model performed better in 63% (5/8), worse in 25% (2/8), and equal in 13% (1/8); median gain in AUC was + 0.025.

**Conclusions:**

Overall, DMs outperformed GMs but in 26% of the studies, DMs did not outperform GMs.

**Supplementary Information:**

The online version contains supplementary material available at 10.1186/s41747-023-00325-0.

## Key Points

- Deep learning (DL) models outperform generic models often but only in 3 out of 4 studies.
- Fused models can improve over the generic and DL models.
- Data leakage, model selection and optimisation, and publication bias could affect the comparison between generic and DL models.
- It is worthwhile to explore both modelling strategies in practice.

## Background

The application of machine learning (ML) to radiological imaging is a fairly old idea and can be traced back at least to the 1970s [1]. There are many benefits to such an approach. First, it can be noninvasively applied to various tasks like diagnosis, classification, and prognosis [2–4]. Second, because imaging potentially contains more information than humans can process, ML can exploit it systematically, which could lead to superhuman performance. Finally, it allows for automation of time-consuming tasks, saving human resources for other essential duties.

**Fig. 1:**
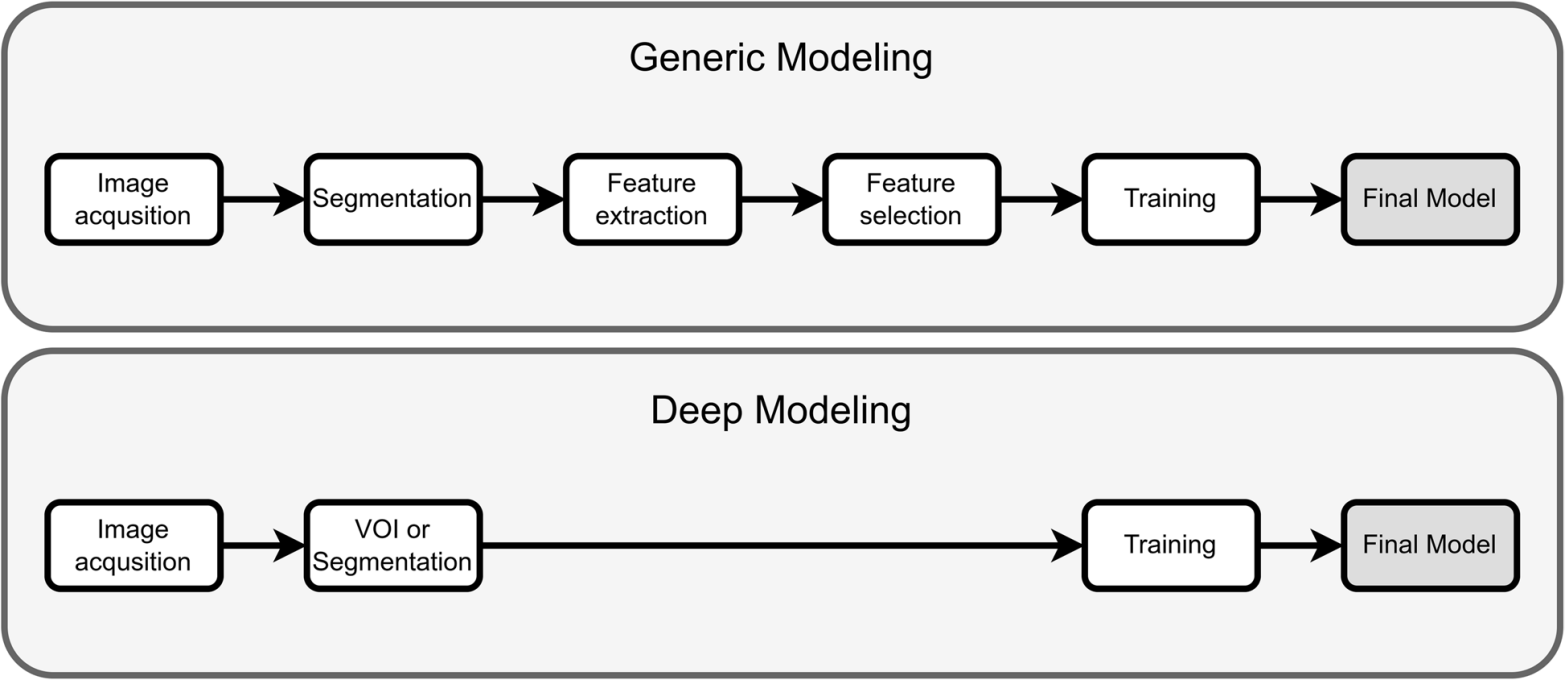
Generic and deep modelling applied to radiomics

While this idea was explored in several studies in the 1990s and 2000s under the heading of texture analysis [5–7], it prominently resurfaced only in 2012, when it was coined “radiomics” in a seminal paper by Lambin et al. [8]. A classic ML pipeline applied to radiomics consists of several steps (Fig. 1) [9]. It was shown that radiomics can lead to accurate models [10–12].

Despite its benefits, there are also disadvantages to this approach. A key problem is the segmentation of pathologic findings, which is often tedious work. Even though some (semi)automated solutions exist [13], they are not always ready for diagnostic purposes. A second disadvantage is the set of features that are used to characterise the pathologies quantitatively; these are defined generically by the use of explicit formulas and are often derived from morphological, statistical, and textural properties [14]. We call such features “generic” since they are not specific to the problem at hand and could thus be unable to capture all information present in the data. Hence, models based on generic features, *i.e.*, “generic models” (GMs), could perform suboptimally. In contrast, models could learn features directly from datasets during training without requiring explicit formulas. This approach, however, requires different methods.

Deep learning (DL), which is a subarea of ML based on neural networks, could be able to solve the disadvantages of generic models. While the main idea dates back to the first ML concepts in the 1950s [15], and networks already have been applied to radiological data in the 1990s [16], only recently, new techniques and increased computation power allowed these networks to solve many interesting problems that were previously thought to be hard.

Compared to generic modelling, the DL pipeline involves fewer steps (Fig. 1), as deep networks can learn directly from images without the explicit need for any segmentations. Equally important, they can learn predictive features independently during training, bypassing the need for explicit feature definitions. We will refer to models that use features learned implicitly from data by DL as “deep models” (DMs). Because deep models are adapted to data, it is reasonable to expect them to yield better results than GMs.

Yet, there are drawbacks to deep modelling. Since the networks are not given previously defined features, they usually need more data to find predictive patterns. However, larger sample sizes are often unavailable in a radiomic context. In addition, the reproducibility and generalizability of deep networks are unclear since they are known to be sensitive to the initial weights and might behave erratically [17–19]. Both might render any advantage of deep modelling against generic modelling void.

Nonetheless, since DL methods have been applied successfully in many fields, they are generally considered to be superior to generic modelling in radiomics, even though large-scale experiments that analyse this question in-depth are currently missing. Indeed, some radiomics studies using DL report higher predictive performance than generic modelling [20, 21], but other studies report no improvements [22, 23].

This observation raises the question of whether deep modelling is truly superior. Therefore, in this review, we examined studies that directly compare deep and GMs to determine whether a difference in predictive performance can be seen. We also discuss the influence of a few modelling decisions and potential biases for differences in performance between the two models.

### Modelling strategies

Generic and deep modelling have many processing steps in common (Fig. 1). The main difference relates to the generation of features. While in generic modelling standardised features are extracted from the imaging data, deep modelling can employ a wide range of network architectures to find optimal features.

### Generic modelling

The overall pipeline of generic modelling is rather standardised (Fig. 1) [3, 24]. Starting from image acquisition, a key step is to segment the pathologies. This is necessary to focus the computation of the features on the relevant area of the imaging. Features are then extracted from the volumes and are used to train a classifier.

In all these steps, certain choices have to be performed. For example, the imaging data is first discretised to avoid that the extracted features depend too much on the inherent noise [25, 26]. This discretisation proceeds either by binning the data into bins of fixed widths or a predefined number of bins, and it is unclear which of the two approaches works better for a given dataset. From these discretised images, many features, like maximum, minimum, mean, and variance, are computed using explicit formulas, which characterise the image in a specific way. However, since it is not known beforehand which features will be predictive, many irrelevant and redundant features will be present. Feature selection methods are applied to remove these, and the remaining features are then processed by a classifier [27].

While the choice of the feature selection method and classifier is central to obtaining a high-performing model, in our study, we took the GM as a baseline and therefore only considered decision choices regarding the deep modelling.

### Deep modelling

Deep modelling can be more complex than generic modelling, as neural networks can be built with many different topologies and architectures. A description of deep networks is beyond the scope of this review; details can be found in the literature [28–30]. In a nutshell, a deep network can be described as a model with multiple layers of neurons connected by weights. These weights are used to transform input data into output data; for example, a scan depicting a tumour could be transformed into a prediction of its malignancy. The weights, therefore, are central since they determine the network’s output. Training of a network can then be understood as a process to optimise the weights so that input data is transformed to the corresponding label.

However, deep networks are parameterised by a vast number of weights, numbering in the millions. Thus, a sufficiently large number of training samples is required for successful training, making them unsuitable per se in areas such as radiomics, where only limited sample sizes are available. Pretraining is a commonly used trick to get around this problem, where the network is trained on data from another domain. The hope is that by pretraining, the weights will be in a near-optimal state so that for successful training of the problem at hand, fewer samples are necessary.

Pretraining, however, cannot be directly applied to radiomic data since many pretrained networks were trained on photographs and can, therefore, only process two-dimensional (2D) data, while radiomic data is often three-dimensional (3D). A solution would be to process the radiomic data slice by slice, but in this approach, the spatial context is lost, and the network’s performance will be suboptimal. On the other hand, employing a 3D network is also difficult because of the low sample sizes; pretrained 3D networks are also currently unavailable, further deepening the problem. Therefore, a critical choice in developing a DM is whether the network should be 2D or 3D and whether pretraining should be used.

Networks can also be trained in an end-to-end fashion or used as feature extractors. In the end-to-end case, the network is trained and used as a whole. In contrast, when used as a feature extractor, features are extracted at an intermediate layer of the network and then processed using classical machine learning methods. The advantage of this approach is that other techniques can be used for classification, possibly improving the overall performance.

### Fused models (FMs)

Models that fuse generic and deep models are of particular interest, as they should be able to harness the advantages of both modelling strategies and lead to yet higher predictive performance since fusing works similarly to a small ensemble [31]. The fusion can take place on multiple levels; the most basic approach is to take the average of the output of both models. If the network is used as a feature extractor, another approach would be to merge generic and deep features and apply feature selection and classification methods to this merged feature set [32]. Alternatively, the generic features can be added directly to the network, so it can utilise them during training [33]. More complicated fusion methods are also possible [34, 35], but because generic features are rather fixed, the options mainly affect the neural network’s architecture.

Fusing does not come without disadvantages; the FM depends on the GM, which in turn usually depends on fine segmentation. Therefore, the key advantage of deep modelling is lost in FMs. In addition, since the FM will have more hyperparameters, the risk of overfitting is higher [36].

### Literature review

We conducted a literature search to find studies comparing the two modelling approaches to gather evidence on their relative performance.

### Search protocol

We identified publications published before 2022 by querying PubMed and Embase databases using the keywords “radiomics” and either “deep learning”, “deep neural”, or “deep network” (Supplementary material 1).

### Study selection

Abstracts of the publications were first screened; all studies which were not original research were removed. Studies were excluded that either did not report on a binary outcome or were not using 3D data. Studies were not considered eligible if as follows: (a) no GM was trained or reported; (b) no DM was trained or reported; (c) only a FM was trained or reported; (d) deep learning was not used for modelling; (e) no area under the curve (AUC) was reported; and (f) the validation scheme was unclear. In addition, AUCs from segmentation or survival tasks were not included in our study since these AUCs differ; for example, a segmentation is a low-dimensional problem, which has different statistical properties than the high-dimensional problems that usually occur in radiomic studies with a binary outcome.

### Study questions

The main question of this study was *whether models based on deep modelling perform better than GMs in terms of predictive performance, measured by AUC*. In addition, we aimed to answer the following questions: (a) Do DMs perform better on external data than GMs? (b) Do FMs perform better than either the GMs or DMs? (c) Do 3D network architectures outperform 2D networks? (d) Does pretraining help to improve predictive performance? (e) Does the use of deep networks as feature extractors increase the gain in AUC over end-to-end learning? These questions concern only deep modelling since we consider GMs as the baseline.

### Data extraction

We listed the sample size and the validation scheme used for each study. We then extracted the predictive performance of the generic, deep, and FMs in the internal and external cohorts. Internal data refers here to data collected at the same hospital or centre; external data is those gathered at a different hospital or centre. Predictive performance was extracted as mean and 95%-confidence intervals or converted from standard deviation where possible [37]. The difference between the AUC of the generic, deep, and FMs was then computed and graphically displayed separately for models tested on internal and external data.

### Statistics

Descriptive statistics results were reported as median. Statistics were computed using Python 3.10.4.

## Results

### Literature research

**Fig. 2:**
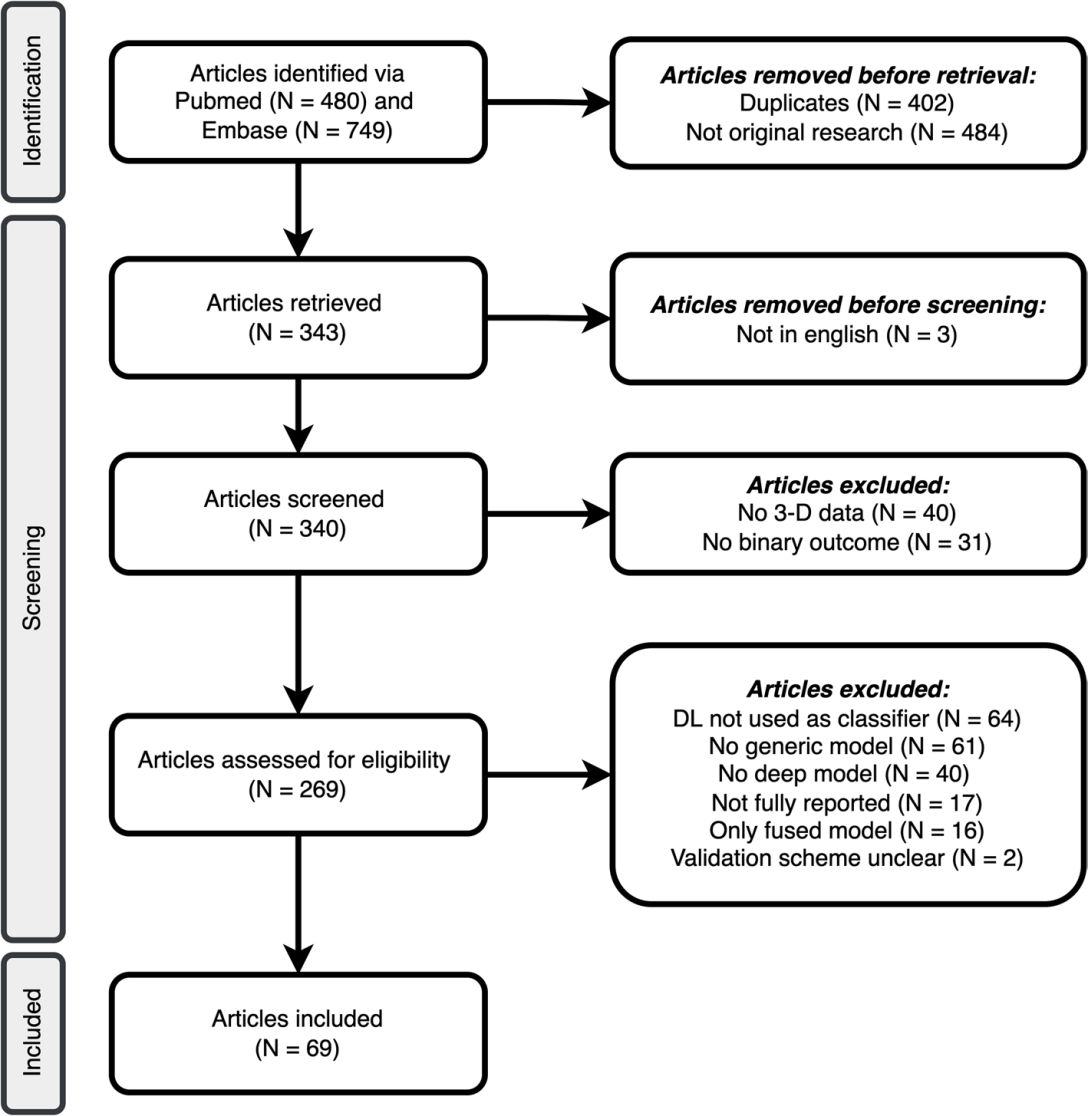
Inclusion and exclusion flowchart

**Fig. 3:**
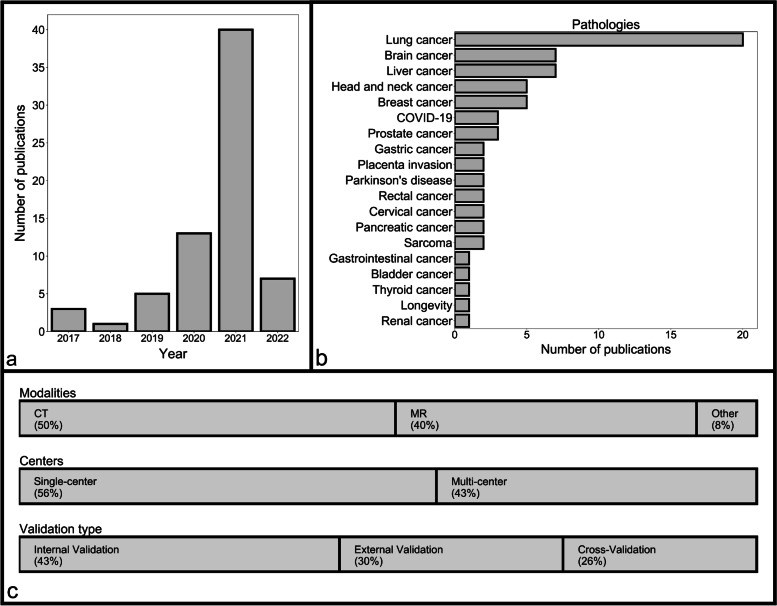
Characteristics of the included studies

Of 1229 records, 69 studies were included in the analysis (Fig. 2, Supplementary material 2). An overview of all included studies is presented in Table 1. Publication dates ranged from 2017 to 2022, with most studies conducted in 2021 (Fig. 3); no relevant study was found before 2017, whereas a few studies were available in 2021 ahead of final publication and therefore had a publication date of 2022.

**Table 1:**
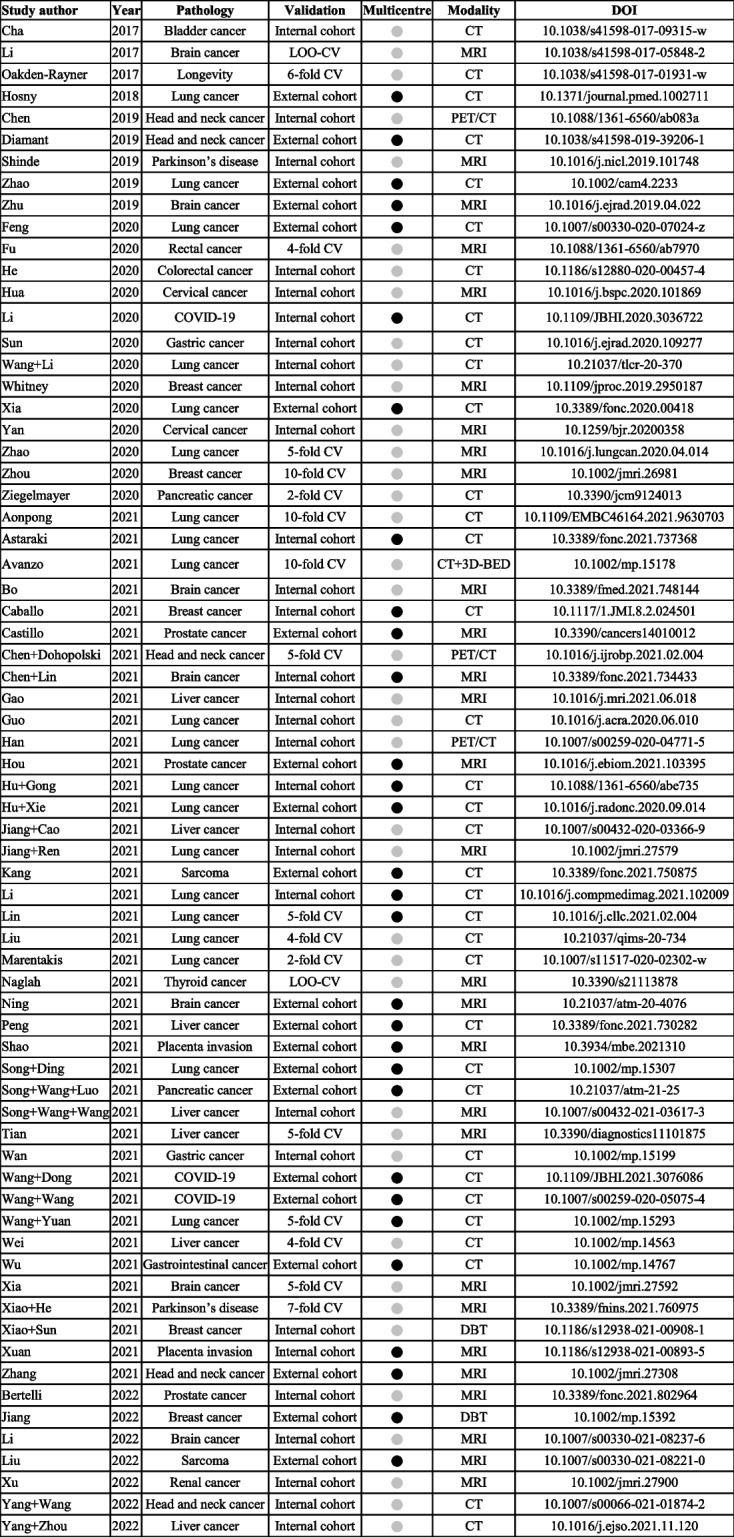
Overview of the characteristics of all included studies

If multiple outcomes were analysed in a study, only the outcome with the highest area under the curve was reported. Note that Li (2020), Astaraki (2021), Caballo (2021), Chen + Lin (2021), Li (2021), Lin (2021), and Xuan (2021) used data from external sites; however, the data were merged and randomly split before modeling. Therefore, the results were considered to be internally validated, not externally.  yes,  no, *3D-BED* Three-dimensional biologically effective dose, *COVID-19* Coronavirus disease 2019, *CV* Cross-validation, *CT* Computed tomography, *DBT* Digital breast tomosynthesis, *LOO-CV* Leave-on-out-cross-validation, *MRI* Magnetic resonance imaging, *PET* Positron emission tomography

### Study characteristics

Except for one study [38], all were retrospective in nature. Studies varied greatly in their sample size (Table 2). In the studies that used an independent internal cohort, the median sample size of the training cohorts was 196 (range 41–1,455). In contrast, in the studies that employed cross-validation only, without an independent internal cohort, the training sample size was even smaller (133, range 43–1,426). The validation cohorts were also smaller than the training cohorts; the internal cohorts had a median size of 73 samples (range 18–535), and the external cohorts had 94 samples (range 18–388).

**Table 2:**
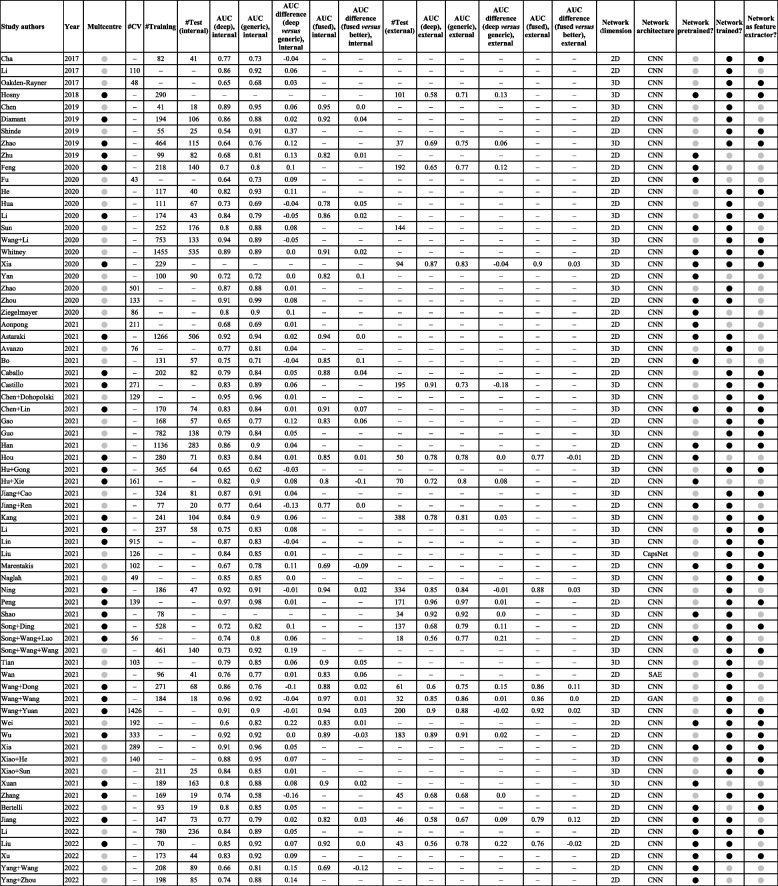
Overview of the predictive performance reported in the included studies

If only a cross-validation was performed without an independent internal validation cohort, the number of training samples across all folds is reported the #CV column. Otherwise, if internal validation cohort was available, then the number of training and validation samples are reported in the #training and #test column. Note that Li (2020), Astaraki (2021), Caballo (2021), Chen + Lin (2021), Li (2021), Lin (2021), and Xuan (2021) used data from external sites; however, the data were merged and randomly split before modeling. Therefore, the results were considered to be internally validated, not externally. Note also that Hu + Gong (2021) and Song + Wang + Luo (2021) use a U-Net, which is a generative network.  yes,  no, *2D* Two-dimensional, *3D* Three-dimensional, *CapsNet* Capsule neural network, *CNN* Convolutional neural network, *GAN* Generative adversarial network, *SAE* Sparse autoencoder

Nearly all studies related to tumours (88%). More studies used computed tomography than magnetic resonance imaging (50% *versus* 40%) and were conducted on only one site (56%); accordingly, most studies either used an internal validation cohort (43%) or applied cross-validation (26%).

### Predictive performance

**Fig. 4:**
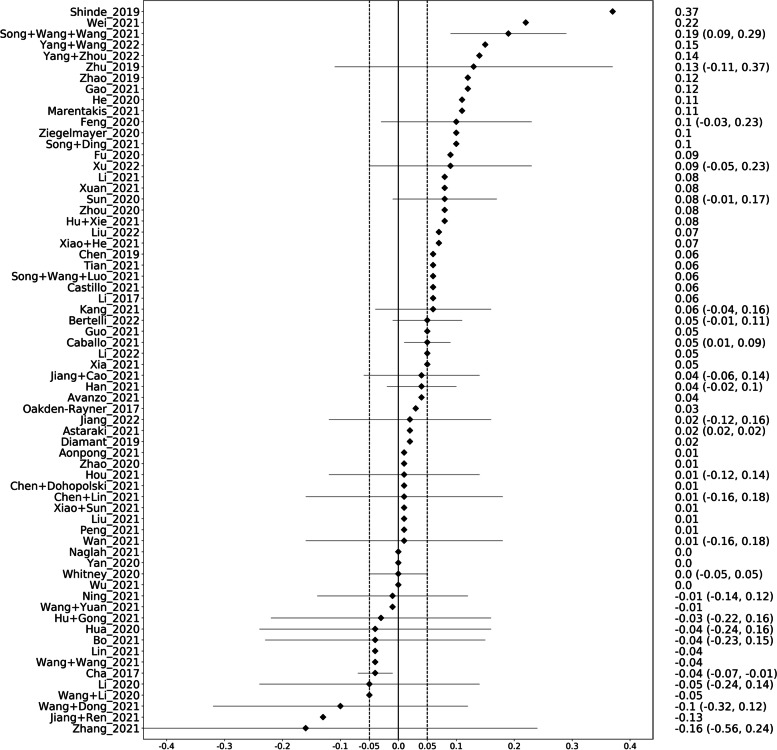
Graphical display of the performance differences between the generic and deep models on the internal validation sets. On the right, the difference in area under the curve together with the 95% confidence interval is given. A positive difference means that the deep model performed better than the generic model

Comparing the performance of generic and deep modelling on the internal validation sets, in 74% (49/66), the DMs performed better than the GMs and vice versa in 20% (13/66) of the cases (Fig. 4). In 6% (4/66), there was no difference. The median difference in AUC was 0.045.

**Fig. 5:**
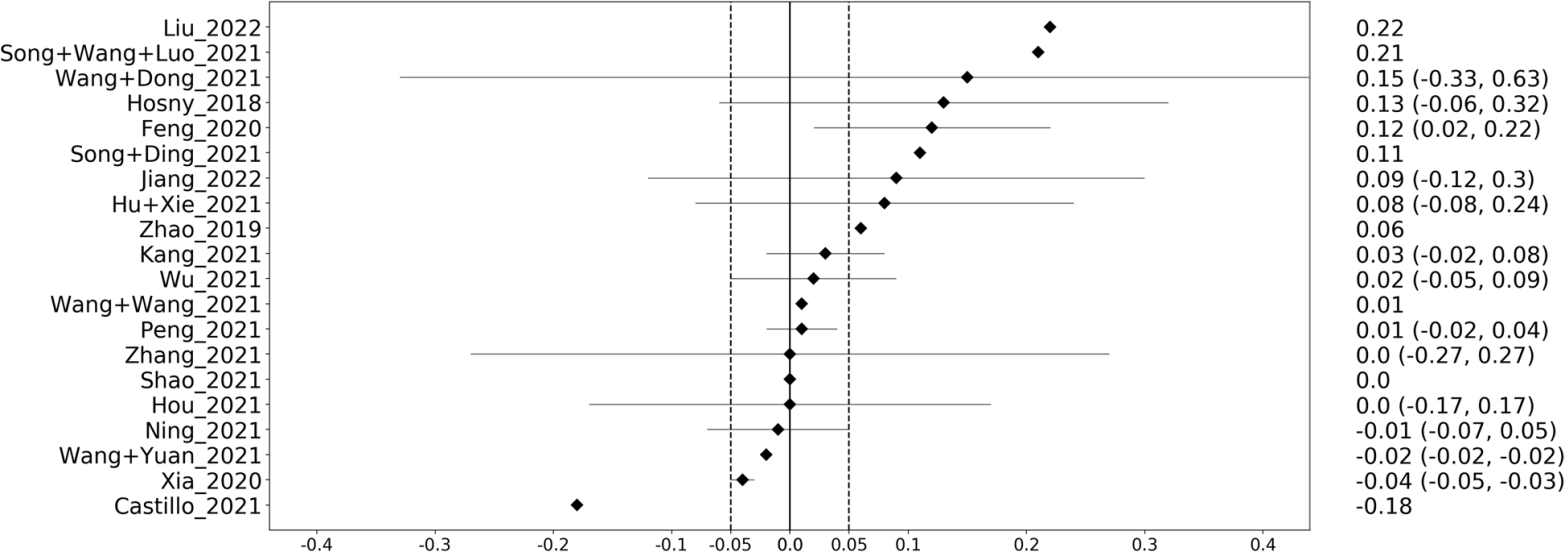
Graphical display of the performance differences between the generic and deep models on the external validation sets. On the right, the difference in area under the curve together with the 95% confidence interval is given. A positive difference means that the deep model performed better than the generic model

On the external validation sets, the DMs were better in 65% (13/20) of the cases and the GMs in 20% (4/20) cases (Fig. 5). In three cases (15%), no difference was seen. The median difference in AUC was 0.025.

**Fig. 6:**
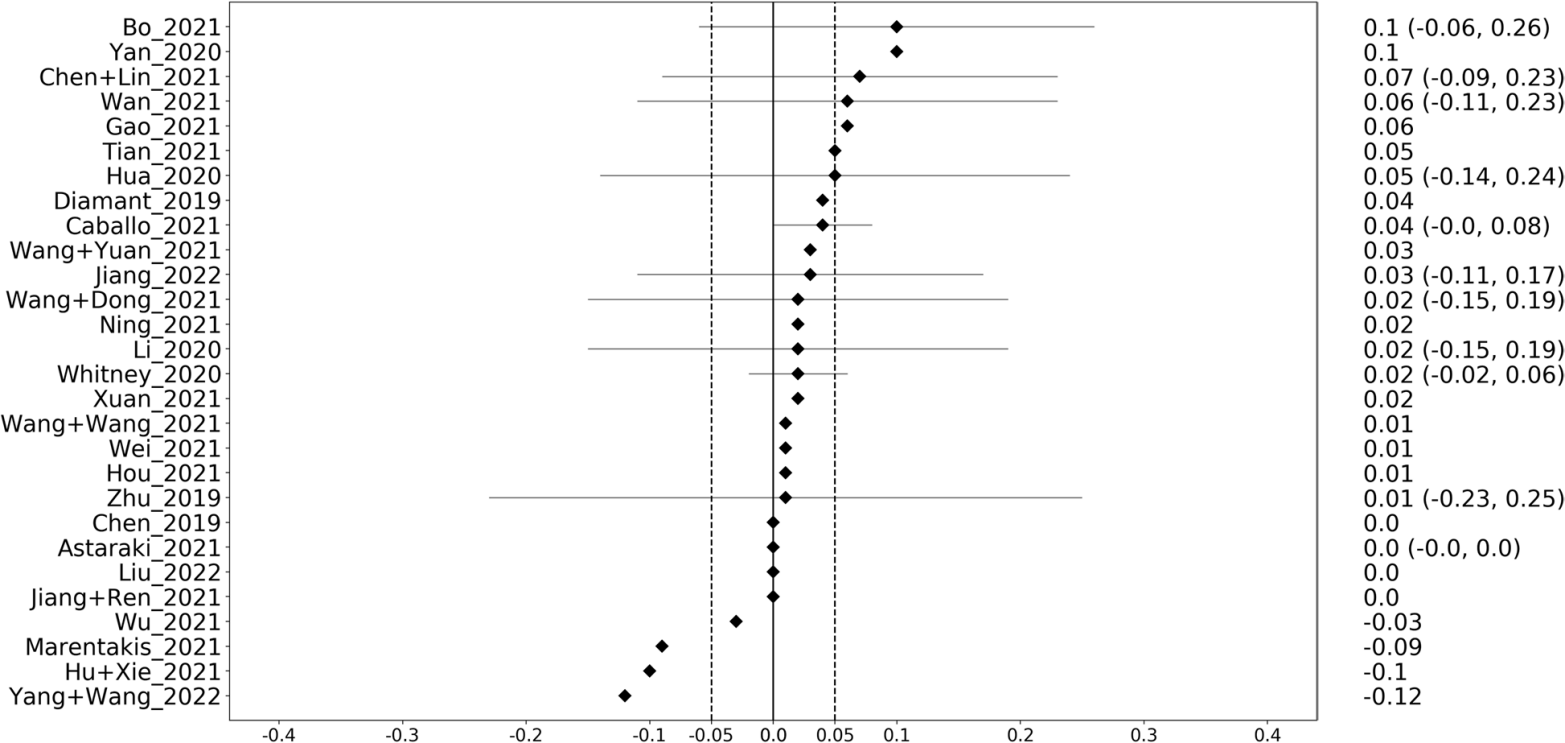
Graphical display of the performance differences between the fused and the better of generic and deep models on the internal validation sets. On the right, the difference in area under the curve together with the 95% confidence interval is given. A positive difference means that the fused model performed better than the deep and the generic models

**Fig. 7:**
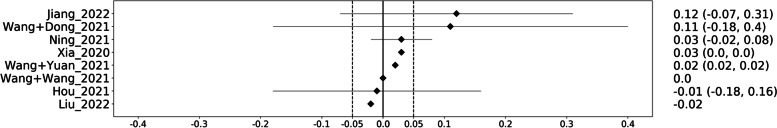
Graphical display of the performance differences between the fused and the better of generic and deep models on the external validation sets. On the right, the difference in area under the curve together with the 95% confidence interval is given. A positive difference means that the fused model performed better than the deep and the generic models

A similar picture emerged when considering the FMs. On the internal validation sets, the FMs outperformed the better of GMs and the DMs in 72% (20/28), while it was worse in 14% (4/28) and equal in 14% (4/28) (Fig. 6). The median performance gain in AUC was + 0.02. On the external validation sets, the FM performed better in 63% of the cases (5/8), worse in 25% (2/8), and equal in a single case (13%) (Fig. 7). The median gain in AUC was + 0.025.

### Characteristics of deep modelling

Nearly all DMs were based on convolutional neural networks (CNN) (96%, 66/69), with only three exceptions that used either a capsule network [39], a generative adversarial network [40], or a sparse autoencoder [41]. Note that two studies employed a U-Net, which is generative CNN [42, 43]. Most DMs used 2D architectures (58%, 40/69), while pretraining was performed less frequently (45%, 31/69). In 78% (54/69) of all studies, the network was trained using the data; in the remaining 22% (15/69), no training was performed. The network predictions were used directly in 55% (38/69) of the cases, while in 45% (31/69), the network was used as a feature extractor.

Regarding the characteristics of the deep networks, 2D networks performed better than 3D networks when comparing both to the GMs: the median performance gain in AUC in the internal cohorts for 2D networks was, on average, + 0.05 and for 3D networks + 0.02 (Table 3). A slightly higher gain could be seen in the external cohorts (+ 0.08 *versus* + 0.0). Furthermore, pretraining yielded a higher performance gain (internal cohorts: median AUC + 0.07 *versus* + 0.02; external cohorts: + 0.09 *versus* + 0.01). Using the network in an end-to-end fashion or as a feature extractor did not make a clear difference in the internal cohorts (median AUC + 0.05 for both). The situation was different in the external cohorts (+ 0.02 *versus* 0.09).

**Table 3:** Overview of the influence of network characteristics on the predictive performance relative to generic modelling

		Internal validation cohorts				External validation cohorts			
	Network characteristic	Median gain in AUC	Better	Equal	Worse	Median gain in AUC	Better	Equal	Worse
Dimension	Two-dimensional	+ 0.05	78% (31/40)	8% (3/40)	15% (6/40)	+ 0.08	82% (9/11)	0% (0/11)	18% (2/11)
	Three-dimensional	+ 0.02	69% (18/26)	4% (1/26)	27% (7/26)	+ 0.00	44% (4/9)	11% (1/9)	44% (4/9)
Weights	Pretrained	+ 0.07	86% (24/28)	7% (2/28)	7% (2/28)	+ 0.09	67% (6/9)	22% (2/9)	11% (1/9)
	Trained from scratch	+ 0.02	66% (25/38)	5% (2/38)	29% (11/38)	+ 0.01	64% (7/11)	9% (1/11)	27% (3/11)
Approach	End-to-end	+ 0.05	72% (26/36)	8% (3/36)	19% (7/36)	+ 0.02	60% (6/10)	10% (1/10)	30% (3/10)
	Feature extractor	+ 0.05	77% (23/30)	3% (1/30)	20% (6/30)	+ 0.09	70% (7/10)	20% (2/10)	10% (1/10)

The median gain in area under the curve (AUC) was calculated as the difference in performance from the generic models across all studies that used a network with the corresponding feature. Similarly, the “better”, “equal”, and “worse” columns denote the number of studies that reported better, equal, or worse AUC of the deep model (with the corresponding feature) compared with the generic model

## Discussion

Generic and DL models are currently in use in radiomics, but the difference in predictive performance has not yet been analysed across studies. Therefore, we reviewed studies that used both modelling strategies and identified 69 studies that provided a direct comparison.

### Predictive performance

Overall, there was a significant advantage of deep over generic modelling, as evident by an increase in median AUC of + 0.045 in the internal cohorts. However, in 26% of the studies, no increase was visible. It is unclear whether this depended on the data or if the modelling was not performed as well as it could have been. A lower difference was found in external cohorts (AUC + 0.025), indicating that DMs perform on external data at least as good as GMs. Fusing the two modelling approaches had similar gains (AUC + 0.02 and + 0.025). Since the overall number of studies with a FM was smaller, the effect must be read cautiously.

### Characteristics of DL modelling

As expected, network architectures derived directly from CNN were used nearly exclusively, and other architectures were vastly underexplored. Therefore, we considered three deep modelling choices that are relevant for CNNs: the dimensionality of the network, the use of pretrained weights, and end-to-end training. It turned out that on average, 2D networks performed better than 3D networks when compared to the GMs (AUC + 0.05 *versus* + 0.03); nonetheless, the median sample sizes of the training sets were higher for 3D networks (224 *versus* 166 samples), showing that most studies preferred 3D networks when sample sizes were higher. Pretraining did yield a higher performance gain (AUC 0.07 *versus* 0.02). Finally, using the network in an end-to-end fashion or as a feature extractor did not make a difference (AUC + 0.05 *versus* + 0.05).

These observations must be taken with some caution since they only reflect an average tendency. For example, pretraining did not yield superior results in some studies [22, 44, 45]; similarly, 3D networks can perform better [46].

### Sample size

The data sets used were relatively small on average, as reflected in the median sample sizes of the training cohorts (*n* = 196). However, the sample sizes of the test cohorts were even smaller (*n* = 73 and *n* = 94). Thus, caution should be exercised when using such small cohorts to demonstrate that one modelling approach is statistically better than another. Because a rule of thumb derived from simple statistical distributions requires at least 30 samples per group to establish a statistical difference [47, 48], the sample sizes of the studies appear to be too small.

### Validation and generalisability

Many of the included studies used an internal test cohort instead of applying cross-validation. However, testing on a single split can be unreliable [49], and since cross-validation can be understood as applying systematically repeated splits, it should be preferred. Most of the studies also did not test their model on external data, since setting up a large-scale multicentre study has a high organisational cost. Their generalisability, measured as performance on external data sets, is therefore unclear. In addition, since nearly all studies were retrospective in nature, their clinical applicability [10] was not tested.

### Sources of bias

Several sources of bias exist which can impede a fair comparison. These include biases in modelling and study quality, interpretation, and publication.

### Data leakage

In both modelling strategies, data leakage can occur; for generic modelling, a few studies seemed to apply the feature selection to all data before applying cross-validation and do not test their model on a fully independent cohort [50]; however, this can lead to a strong positive bias [51]. The same error can occur for DMs if they are used as feature extractors. For DMs, it is also conceivable that some studies misused the validation cohort for testing during training, which would also lead to a positive bias. These problems cannot be detected from the studies, adding complexity to a direct comparison.

### Interpretability

In the literature, generic features are thought to be more interpretable by a human reader than deep features [14]. Therefore, a potential trade-off occurs when using deep features, since a gain in predictive performance would come with a loss in interpretability. This trade-off cannot be directly quantified since it is unclear how to measure “interpretable”. While some generic features like volume and mean intensity have a clear meaning, other features like “Wavelet-HLH_glrlm_RunVariance” are inherently obscure. Nevertheless, the characteristics of the generic features utilised by the model can potentially be used in further experiments to demonstrate a biological correlation, which is not directly possible for deep features. It must be noted, however, that statistically equivalent models may select quite different features [52].

### Bias in modelling

For both modelling strategies, there is a certain bias concerning the used methods. In general, it is unknown a priori which methods will perform best; therefore, it is best practice to test multiple methods. For example, feature selection is crucial in higher dimensions, and different choices can lead to models with vastly different performance [27, 53, 54]. Yet, some of the included studies only consider a single feature selection and classification method for generic modelling [55]; if this method is not adequate for the data, it can lead to an underperforming model and would introduce another bias. Other studies extract only a few features [56] which can also result in a potential loss in performance [57]. DMs can also suffer from such a bias since nearly all studies used a CNN architecture; it is conceivable that other network architectures could perform better [58].

In both modelling strategies, hyperparameters, which are parameters that are not learned during training, need to be selected. This is critical for many models; for example, a network can only perform well if the learning rate is chosen properly. But since tuning hyperparameters is computationally expensive, hyperparameters are often left at their default values, which can lead to degraded performance. This can be problematic if tuning is only applied to one of both models; perceived improvements would be spurious since the comparisons could not be regarded as fair.

Such unfair comparisons might happen more often than expected because many papers aim to show that DMs can yield higher performances than GMs. Therefore, it might happen unintentionally that most efforts will be put into developing the DM, whereas less effort is put into the GM. This is especially true if the DM initially performs worse than expected. In this case, deep modelling might be continued until a better-performing model is found.

### Study quality

A well-known problem in radiomics concerns the quality of studies, which can lead to a lack of reproducibility. Although guidelines exist [59], and a quality score specific to radiomic papers has been introduced [9], many papers still need to adhere to this guideline [60]. Thus, the overall quality of studies may be another bias factor that needs to be considered.

### Publication bias

On top, publishing negative results is still not common. Thus, if studies are undertaken with the hope that the DM improves over the GM, but fail to show this, the results might not be reported. Furthermore, a widely underperforming DM might also be not reported since there is a certain risk that training was performed incorrectly; this is true for the GMs to a far lesser extent since the training is less complex and there are more user-friendly ML frameworks [61, 62].

### Recommendations

Given these results, we recommend not limiting oneself to a GM or a DM but computing both. Care should be taken that the GM is not neglected, but that the full range of methods and parameters is tested. Deep modelling with pretrained 2D networks based on CNN architectures is advisable, although, if permissible, custom 3D network architectures should also be tested. Finally, it also seems to make sense to test a FM as it can improve the predictive performance even further.

### Limitations

Our study has a few limitations. First, a direct comparison between GMs and DMs is influenced by many factors, for example the preprocessing and harmonisation of the images and the choice of segmentations, that is, whether they include tissue beyond the pathology. However, to keep things simple, we only considered a few factors for deep modelling and none for generic modelling, since we regarded these as a baseline. We also only considered AUCs, the most often used measurement, though other measures like sensitivity and specificity are often equally important. In addition, several papers report on multiple methods and outcomes. In these cases, we selected the model or outcome with the highest AUC (regardless whether it was obtained in the training or test cohort). These choices could have potentially introduced a bias into our study.

## Conclusion and future directions

In this review, evidence has been found that deep modelling can outperform generic modelling. However, since this is not always the case, both generic and deep modelling should be considered in radiomics. Even though our results showed that DL outperforms generic modelling by some margin, the comparison was only indirect. A large benchmark study involving several datasets would lead to a better understanding of the modelling strategies and yield more precise recommendations. This would include studies on the reproducibility of deep features since these were only performed for generic features yet [63, 64]. DL also has many more applications in radiomics that need to be explored in detail. For example, it can be used as an image-to-image transformer [65] and for automated segmentations [66]; these possibilities are orthogonal to both modelling strategies and could improve both.

## Supplementary Information

**Additional file 1.**

**Additional file 2.**

## Data Availability

All data generated or analysed during this study are included in this published article (and its supplementary information files).

## Abbreviations

2D: Two-dimensional
3D: Three-dimensional
AUC: Area under the curve
CNN: Convolutional neural network
DL: Deep learning
DM: Deep model
FM: Fused model
GM: Generic model
ML: Machine learning
