# Supplementary material for: Are deep models in radiomics performing better than generic models? A systematic review
Source: Eur Radiol Exp. 2023 Mar 15;7:11. doi: 10.1186/s41747-023-00325-0 (PMC10014394; doi:10.1186/s41747-023-00325-0)
Supplement: Supplementary file 1 — Additional file 1. [file 41747_2023_325_MOESM1_ESM.pdf]

SUPPLEMENTARY MATERIALS

Are deep models in radiomics performing better than generic models?

A systematic review

Search Strategy

The search query used on PubMed was:

[("radiomics"[Title/Abstract] AND ("deep neural"[Title/Abstract] OR "deep learning"[Title/Abstract])) AND (2016:2021[pdat]))].

From the search results, we removed those that were not written in english, all reviews, letters, and other non-original work.

For Embase, the search query was similar:

'radiomics' AND ('deep learning' OR 'deep neural' OR 'deep network') AND [2016-2021]/py
AND ('article'/it OR 'article in press'/it OR 'chapter'/it OR 'conference abstract'/it OR 'conference paper'/it OR 'preprint'/it)

Note that changing the date span to include articles before 2016 did not change the search, in other words, there seemed to be no radiomics papers including any kind of deep learning before 2016.

Reporting

- If multiple AUCs were reported (for example, if multiple models were tested or models were tested on multiple test sets), the combination with the highest AUC was selected.
- If only a cross-validation (CV) and no internal test set was used, then we reported this model as CV even if afterwards an external model was trained. However, if CV is applied, but upfront an internal test set was split off, then the CV results are not reported, since they could be more biased than the results of the test split; the sample size for the CV was then reported as training size.
- Sample sizes are given as the number of patients, even if the training proceeds with lesions or image slices, where a single patient can have multiple samples. However, not in all studies the exact patient numbers were given; in these cases the reported numbers were given and a comment was made below for each paper.
- A network is called pretrained if any of its parts were pretrained. This could either be external data like ImageNet or the same data, but on a different task like prediction of the segmentation.
- Fused models were only considered if they were similar to the generic and deep model; especially, if clinical variables were used for the fused model, but not for the single models, the fused model was not considered. Fused models also had to contain both, generic and deep features. A model that for example combined deep and clinical was not considered to be a fused model for this review.
- Networks were considered to be 2-D if they did use three or less slices, therefore “2.5D” networks, where three slices are processed at the same time, were subsumed under 2-D.
